# EvoMut: A computational framework for engineering oxidative stability in proteins

**DOI:** 10.1002/pro.70774

**Published:** 2026-09-15

**Authors:** Seyed Shahriar Arab, Chenlin Hsieh, Nathan E. Lewis

**Affiliations:** ^1^ Center for Molecular Medicine University of Georgia Athens Georgia USA; ^2^ Complex Carbohydrate Research Center University of Georgia Athens Georgia USA; ^3^ Department of Biochemistry and Molecular Biology University of Georgia Athens Georgia USA

**Keywords:** evolutionary conservation, oxidative hotspot prediction, oxidative stability engineering, protein engineering, protein oxidation, structural bioinformatics

## Abstract

Amino acid oxidation is a major cause of protein instability and loss of function in therapeutic and industrial settings. Although methionine, cysteine, tryptophan, tyrosine, histidine, lysine, and arginine residues are widely recognized as oxidation‐prone, only a subset of such residues is dominant functional hotspots, and not all are suitable targets for mutation. Identifying these vulnerable, yet engineerable, sites remains a major challenge. Here, we present EvoMut, a residue‐level analytical framework for evaluating both oxidative vulnerability and mutation feasibility. EvoMut estimates oxidation risk by integrating structural features, local functional context, intrinsic chemical susceptibility, and evolutionary conservation. A central feature of the framework is the explicit separation of oxidation risk from mutation feasibility. Specifically, candidate substitutions are evaluated only after high‐risk residues are identified and ranked by evolutionary substitution patterns. Application of EvoMut to multiple proteins, and evaluation with experimental data, showed that oxidation‐prone residues differ markedly in their engineering potential. EvoMut distinguishes residues that are both oxidation‐sensitive and evolutionarily permissive from those that are chemically vulnerable but functionally constrained. By providing residue‐level mechanistic insight, EvoMut offers a practical framework for the rational design of oxidation‐resistant proteins. EvoMut is freely available as a web server at https://proteus.cmm.uga.edu/evomut.

## 1 INTRODUCTION

Oxidative modification is a pervasive phenomenon in protein science and represents a major source of structural instability, loss of enzymatic activity, and reduced functional lifetime in biological, industrial, and therapeutic contexts (Demasi et al., 2021; Rosenberg, 2006; Stadtman, 2006; Wang et al., 2025). Reactive oxygen species (ROS) can damage proteins during production, formulation, storage, and in vivo circulation. Even subtle chemical modifications at specific residues may disrupt local interactions, alter folding behavior, promote aggregation, and ultimately compromise protein function.

At the molecular level, ROS preferentially target amino acids with high redox reactivity. Methionine and cysteine are often the most susceptible residues, and their oxidation can produce methionine sulfoxide or various cysteine derivatives that perturb tertiary structure and functional integrity (Demasi et al., 2021). Aromatic residues, including tryptophan and tyrosine, are also subject to radical‐mediated oxidation that promotes covalent cross‐linking and aggregation (Demasi et al., 2021; Rosenberg, 2006). Beyond these classical targets, histidine, lysine, and arginine residues can also undergo oxidative modification, particularly under metal‐catalyzed oxidation and carbonylation conditions, yielding carbonyl derivatives that alter protein charge and functional properties (Amici et al., 1989). Despite this chemical selectivity, experimental evidence consistently shows that oxidative damage is not uniformly distributed among oxidation‐prone residues within a protein (Gettins, 2002; Griffiths & Cooney, 2002; Taggart et al., 2000).

In many proteins, oxidative impairment can be dominated by a limited number of critical sites, although in other systems the cumulative effect of multiple low‐abundance modifications contributes substantially to functional loss. These residues are not necessarily the most solvent‐exposed or intrinsically reactive in isolation; instead, they occupy positions where structural context, functional coupling, and dynamic accessibility amplify the consequences of oxidative modification. Consequently, simple heuristics such as equating solvent accessibility with high oxidation risk or burial with protection often fail to explain experimental observations (Ozturk et al., 2013; Yang et al., 2012).

Protein engineering strategies aimed at improving oxidative stability have frequently focused on direct substitution of oxidation‐prone residues with less reactive alternatives (Grøn et al., 1990; Khan, 2025; Sáez‐Jiménez et al., 2015). Although such approaches have been successful in specific cases, their outcomes remain highly variable and may lead to reduced catalytic activity, altered specificity, or compromised stability. This variability partly reflects the fact that protein stability and function emerge from coordinated interactions among neighboring residues, and modification of a single site can propagate indirect effects through local structural networks.

Two distinct factors therefore determine whether a residue is a practical engineering target. The first is oxidative vulnerability, that is, the likelihood that a residue will undergo chemical modification in its native structural and functional environment. The second is mutation feasibility, which reflects whether substitution at that position is compatible with structural, functional, and evolutionary constraints. A residue may be highly susceptible to oxidation yet remain functionally indispensable, severely restricting possible substitutions (Ding et al., 2024; Sáez‐Jiménez et al., 2015; Taggart et al., 2000). Conversely, some oxidation‐prone residues may tolerate a broad range of substitutions with minimal functional consequences.

Here we introduce EvoMut, an integrative analytical framework that evaluates oxidative vulnerability and mutation feasibility as related but distinct residue‐level properties. EvoMut estimates oxidation risk through a weighted integration of structural features, local functional context, intrinsic chemical susceptibility, and evolutionary conservation. For residues identified as high risk, the framework further evaluates mutation feasibility using evolutionary substitution patterns and structural contact relationships. By placing candidate substitutions within their broader structural and evolutionary context, EvoMut provides a systematic framework for identifying engineerable oxidation hotspots and guiding the rational design of oxidation‐resistant proteins.

## 2 RESULTS

### 2.1 EvoMut framework and design principles

**FIGURE 1:**
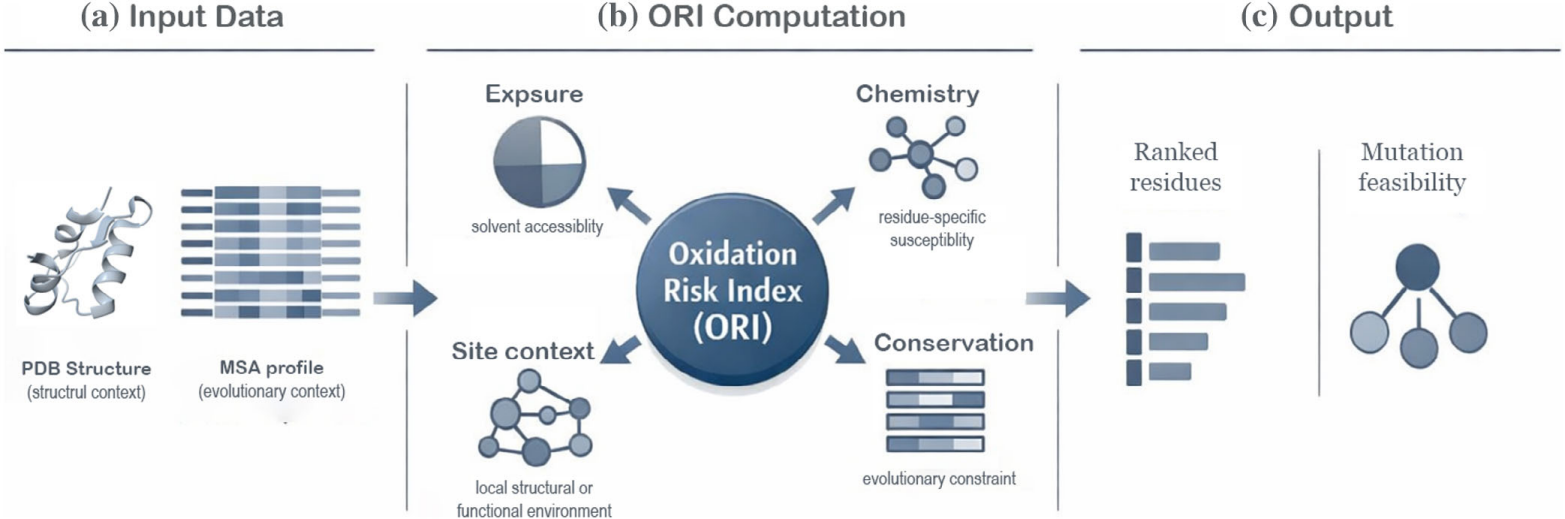
Schematic overview of the EvoMut framework. Protein structures obtained from the Protein Data Bank (PDB) are integrated with evolutionary sequence profiles derived from multiple sequence alignments (XSSP/HSSP). Residue‐level oxidation susceptibility is estimated as a weighted combination of solvent accessibility, intrinsic chemical susceptibility, proximity to functional sites, and evolutionary conservation. Residues with elevated oxidation risk are subsequently evaluated for mutation feasibility using position‐specific evolutionary substitution patterns, enabling prioritized identification of oxidation‐sensitive yet evolutionarily tractable engineering targets.

In rational protein engineering, two fundamental questions frequently arise. First, which residues represent critical hotspots that strongly influence protein stability or function? Second, once such sites are identified, which substitutions can improve the desired property without disrupting structural integrity or biological activity? We developed EvoMut to address these questions in a systematic and sequential manner. The framework separates hotspot identification from substitution assessment and evaluates each stage using complementary sources of information. A schematic overview of the EvoMut workflow is shown in Figure 1.

### 2.2 Identification of critical sites through multi‐factor modeling of oxidation risk

In EvoMut, identification of oxidation hotspots is not based on a single structural or chemical feature. Instead, oxidation risk is modeled as a composite quantity derived from the weighted integration of several independent factors. These include solvent accessibility, intrinsic chemical susceptibility of the residue, proximity to annotated functional sites, and evolutionary conservation of the position. By combining these determinants, EvoMut avoids relying on simplified assumptions such as equating solvent accessibility with oxidation risk. Residues that are partially buried or located within structurally important regions may still emerge as high‐risk positions when their functional context and chemical susceptibility are considered simultaneously.

Evolutionary information is incorporated at this stage with a relatively small weighting coefficient. This design ensures that strongly conserved residues are not overlooked when other factors suggest elevated susceptibility, while preventing conservation alone from dominating the risk assessment.

### 2.3 Stage‐specific use of evolutionary information for substitution assessment

After oxidation‐sensitive residues are identified, EvoMut evaluates possible substitutions for each hotspot. At this stage, evolutionary information becomes the primary determinant of mutation feasibility. Position‐specific substitution patterns derived from multiple sequence alignments are used to define the set of amino acids that are tolerated at each site. This approach restricts the substitution space to variants that are consistent with the protein's evolutionary history and therefore more likely to preserve structural stability and function. Separating hotspot identification from substitution assessment prevents chemically reactive residues from being automatically interpreted as suitable mutation targets and ensures that engineering decisions remain consistent with evolutionary constraints.

### 2.4 Role of neighboring residues and local structural context

Protein stability and function are rarely determined by a single residue in isolation. Instead, local networks of interacting residues collectively shape the structural environment of a site. EvoMut therefore evaluates residues that are spatially adjacent to each identified hotspot. Structural contact analysis is used to identify neighboring residues that may influence local stability or compensate for perturbations introduced by mutation. Evolutionary patterns of these neighboring positions are also examined to assess whether limited adjustments in the surrounding environment may improve the compatibility of a proposed substitution.

This context‐aware analysis allows EvoMut to support both single‐site mutation strategies and targeted multi‐site adjustments when local structural interactions suggest that coordinated changes may be beneficial.

### 2.5 Interpretability in support of engineering decisions

The central design objective of EvoMut is interpretability. Rather than producing opaque predictions, the framework reports residue‐level results that can be traced back to contributing structural, chemical, and evolutionary factors. For each identified hotspot, the contribution of individual components to the calculated oxidation risk can be examined, and proposed substitutions are directly linked to observed evolutionary frequencies. This transparency allows EvoMut outputs to be interpreted as experimentally testable hypotheses that can guide rational protein engineering strategies.

### 2.6 Global identification of oxidation‐sensitive residues

Application of EvoMut to proteins with available structural and evolutionary information revealed that oxidative susceptibility is not uniformly distributed along protein sequences. Instead, a relatively small subset of residues consistently dominates oxidation‐related functional vulnerability. Although several amino acids are chemically susceptible to oxidative modification, only a fraction of these residues emerge as dominant hotspots when structural context and evolutionary constraints are considered simultaneously. In many proteins, multiple oxidation‐prone residues are present, and certain positions can account for a disproportionate share of the observed functional loss; however, in other systems the cumulative effect of multiple low‐abundance modifications distributed across several sites is the primary driver of inactivation (Fuentes‐Lemus et al., 2018; Leinisch et al., 2017; Ozturk et al., 2013). Because most oxidants simultaneously modify several positions, attributing functional loss causally to a single site often requires experimental evidence beyond correlative observations.

Residues identified as high‐risk sites typically exhibit a convergence of several contributing factors. These include intrinsic chemical susceptibility, structural exposure or functional proximity to catalytic regions, and evolutionary patterns that allow limited but plausible substitutions. When these determinants act together, certain positions display markedly elevated oxidation risk relative to other chemically similar residues within the same protein.

This observation highlights the importance of integrative residue‐level analysis. Methods based solely on solvent accessibility or residue chemistry often fail to explain why some oxidation‐prone residues strongly affect protein activity while others remain functionally neutral. The following case studies illustrate how EvoMut distinguishes dominant oxidative hotspots from chemically reactive but functionally marginal residues and how evolutionary substitution patterns further clarify the feasibility of targeted substitutions.

### 2.7 Case study I: Human α_1_‐antitrypsin (A1AT)

Human *α*_1_‐antitrypsin (A1AT) was selected as a benchmark system because its oxidative inactivation has been extensively characterized experimentally. The protein contains 18 residues that are chemically susceptible to oxidation, including nine methionine, six tyrosine, one cysteine, and two tryptophan. Despite this apparent abundance of oxidation‐prone residues, experimental studies have shown that loss of inhibitory activity is primarily driven by oxidation at two specific positions, Met351 and Met358 within the reactive center loop (Silberstein et al., 2018; Taggart et al., 2000; Zhu et al., 2018). In addition, the protein contains 11 histidine, 32 lysine, and 9 arginine residues (chain A, PDB: 1OPH), which represent secondary oxidation targets under specific oxidative conditions such as metal‐catalyzed oxidation and inflammatory oxidants.

**FIGURE 2:**
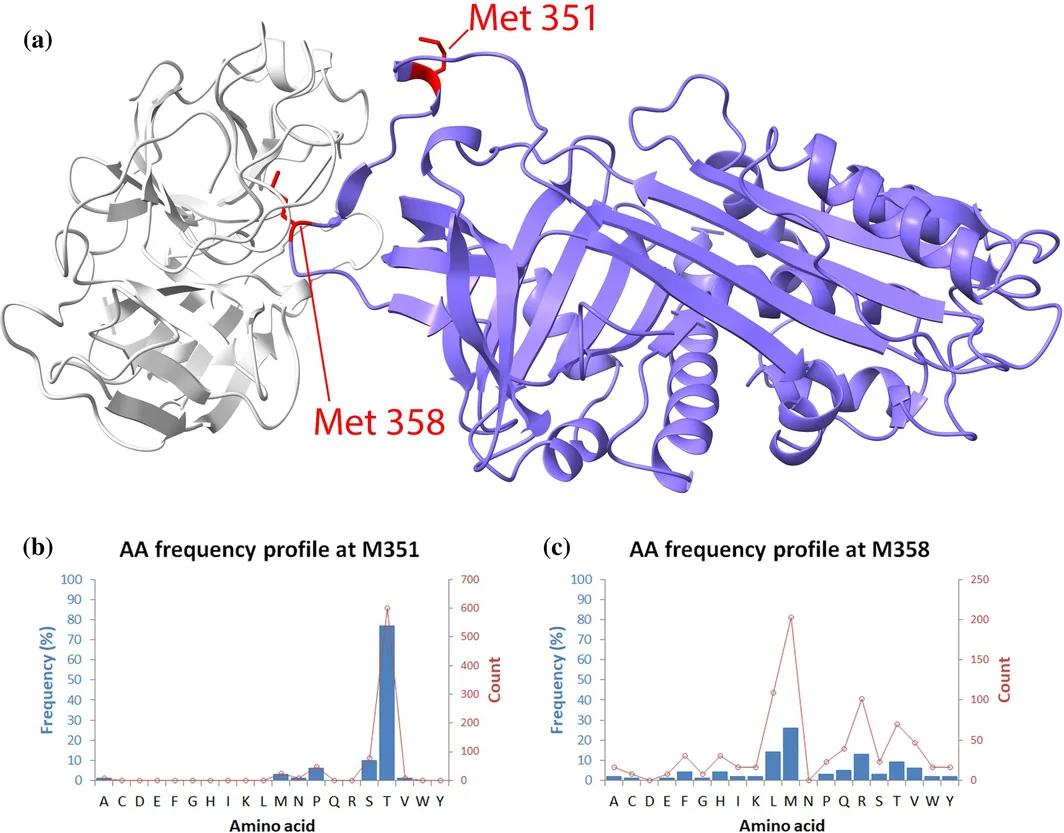
Structural and evolutionary analysis of Met351 and Met358 in A1AT (PDB: 1OPH). (a) Three‐dimensional structure of A1AT highlighting Met351 and Met358 in red within the reactive center loop. (b) Evolutionary residue‐frequency distribution at position 351 showing strong preference for threonine (~77%) with methionine observed at low frequency (~3%), indicating restricted substitution tolerance. (c) Evolutionary residue‐frequency distribution at position 358 showing broader substitution tolerance, with methionine (~26%) and several alternative residues present.

Consistent with these observations, EvoMut analysis identified Met351 and Met358 as the dominant oxidation hotspots in A1AT. Both residues displayed substantially higher oxidation risk scores than the remaining methionines in the protein, forming a clearly separated upper tier in the risk distribution (Table 1). The complete residue‐level ORI values are provided in Supplementary Table S3. Structural mapping further revealed that these residues are located within the reactive center loop, a region that plays a central role in the inhibitory mechanism of A1AT (Figure 2a).

**TABLE 1:** Residue‐level oxidation risk profile for human *α*_1_‐antitrypsin.

Position	WT	WT frq.	ORI
358	M	26	0.84
351	M	3	0.79
226	M	21	0.67
232	C	11	0.58
374	M	3	0.47
242	M	30	0.43
385	M	29	0.43
63	M	69	0.37
238	W	27	0.34
221	M	97	0.33
220	M	98	0.33
160	Y	73	0.22
194	W	99	0.22
187	Y	82	0.19
297	Y	94	0.18
38	Y	89	0.18
244	Y	98	0.17
138	Y	99	0.17

*Note*: Oxidation risk index (ORI) values are reported for all oxidation‐prone residues in the protein sequence. Met358 and Met351 form a distinct upper tier in the risk distribution, reflecting the combined influence of structural context, solvent accessibility, and proximity to the reactive center loop.

Evolutionary analysis provided additional insight into the functional constraints at these two positions. The residue‐frequency distribution derived from sequence alignments indicates that position 351 is strongly biased toward threonine (~77%), with methionine appearing only at low frequency (~3%) (Figure 2b). This restricted substitution pattern suggests that structural or functional requirements limit the range of tolerated residues at this site. In contrast, position 358 displays a broader evolutionary substitution spectrum. Methionine remains present in approximately 26% of sequences, while several alternative residues including leucine, valine, isoleucine, threonine, and glutamine occur at appreciable frequencies (Figure 2c). This pattern indicates a comparatively higher tolerance for amino‐acid replacement at this position.

Together, these results establish a clear residue‐level hierarchy within A1AT. EvoMut identifies Met351 and Met358 as the dominant oxidation‐sensitive hotspots while simultaneously revealing distinct differences in their evolutionary substitution tolerance. This distinction highlights an important aspect of oxidative stability engineering: residues that are similarly vulnerable to oxidation may differ substantially in their suitability as mutation targets.

Beyond ROS‐mediated oxidation, A1AT is subject to additional post‐translational modifications that alter its function through electrophilic rather than oxidative mechanisms. The single cysteine residue C232 undergoes S‐nitrosylation and homocysteinylation in human plasma, modifications that have been linked to changes in inhibitory activity. Lysine residues in A1AT are also susceptible to carbamylation under uremic conditions. These modification pathways are mechanistically distinct from reactive oxygen species chemistry and are not currently modeled within EvoMut, which focuses specifically on oxidation‐sensitive residues under oxidative stress conditions (Lechowicz et al., 2020).

### 2.8 Case study II: Industrial α‐amylases

#### 2.8.1 Alkaline α‐amylase from Alkalimonas amylolytica

The alkaline *α*‐amylase (AmyA) from Alkalimonas amylolytica is an industrial enzyme widely used in detergent and textile applications, where exposure to oxidative agents such as hydrogen peroxide can substantially reduce catalytic performance. In a structure‐guided engineering study by Yang et al., several methionine residues located near the catalytic core were individually substituted with leucine to improve oxidative stability (Yang et al., 2012). Because the experimental study reports residue numbers based on sequence annotation, whereas our analysis uses PDB‐based structural numbering, the corresponding positions are mapped as follows: Met247→349, Met214→316, Met229→331, Met317→421, and Met145→235. For clarity, structural numbering is used throughout the following analysis.

All single Met→Leu variants displayed improved oxidative stability relative to the wild‐type enzyme. Among these mutations, substitution at position 349 (Met247 in the experimental numbering) produced the most pronounced improvement, retaining approximately 72% of catalytic activity after exposure to 500 mM H_2_O_2_.

**FIGURE 3:**
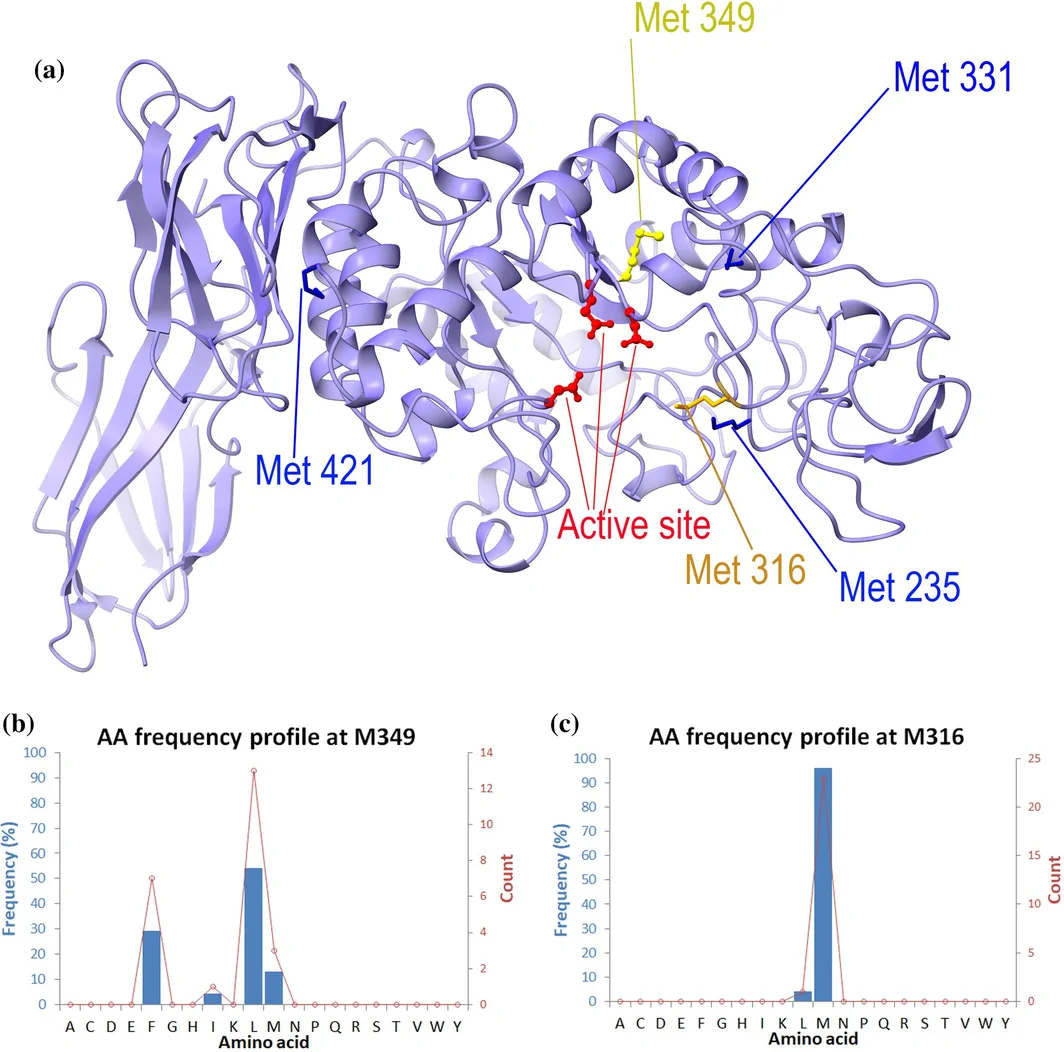
Structural and evolutionary analysis of methionine residues in alkaline *α*‐amylase (PDB: 3BC9). (a) Three‐dimensional structure showing active‐site residues in red. Met349 (yellow) and Met316 (orange) are positioned in proximity to the catalytic region, whereas Met235, Met331, and Met421 are shown in blue. (b) Evolutionary residue‐frequency distribution at position 349, dominated by leucine (54%) and phenylalanine (29%), with methionine present at lower frequency (13%), indicating moderate substitution tolerance. (c) Evolutionary residue‐frequency distribution at position 316, where methionine is strongly conserved (96%) and leucine is observed at low frequency (4%), reflecting strong evolutionary constraint at this position.

Independent EvoMut analysis identified Met349 as the highest‐risk oxidation site among all methionine residues in AmyA (ORI = 0.88). Evolutionary profiling further revealed a restricted substitution space at this position, dominated by hydrophobic residues, with leucine representing the most frequently observed alternative. Consistent with this evolutionary permissibility, EvoMut ranked leucine as the optimal substitution candidate. Structural analysis shows that both residues are located near the catalytic region, yet they differ markedly in their evolutionary profiles (Figure 3a). Methionine is highly conserved at position 316 (~96%), indicating strong evolutionary constraint, whereas position 349 displays lower methionine conservation and a broader substitution spectrum (Figure 3b,c). This contrast suggests that mutation at position 316 may be structurally less tolerated despite its moderate solvent accessibility.

Taken together, these observations illustrate two important principles. First, oxidation vulnerability cannot be inferred from solvent accessibility alone; residues that appear buried may still represent dominant functional hotspots when their structural context and catalytic proximity are considered. Second, evolutionary conservation provides a critical constraint on mutation feasibility. By integrating these factors, EvoMut distinguishes functionally important oxidation hotspots from residues that are structurally constrained but less influential in oxidative inactivation.

The strong agreement between experimental structure‐guided engineering and EvoMut's residue‐level prioritization highlights Met349 as the dominant oxidative vulnerability in AmyA and demonstrates the utility of integrative structural and evolutionary analysis for guiding oxidation‐resistance engineering.

#### 2.8.2 α*‐Amylase AmyC from Thermotoga maritima*

The intracellular *α*‐amylase AmyC from Thermotoga maritima was analyzed to evaluate site‐specific variation in oxidation susceptibility and substitution tolerance at methionine residues that have been experimentally investigated. In a previous mutagenesis study, Ozturk et al. examined four positions (Met43, Met44, Met55, and Met62) and reported that substitution of Met55 with alanine (M55A) produced the most pronounced improvement in oxidative stability and enzymatic performance among the tested variants (Ozturk et al., 2013).

Consistent with these experimental observations, EvoMut analysis ranked Met55 as the most oxidation‐sensitive residue among the examined positions. The calculated oxidation risk index (ORI) was highest for Met55 (0.49), followed by Met43 (0.48), whereas Met62 and Met44 showed slightly lower and comparable values (0.46 and 0.45). These results indicate that position 55 represents the dominant oxidation hotspot within the experimentally investigated set.

**FIGURE 4:**
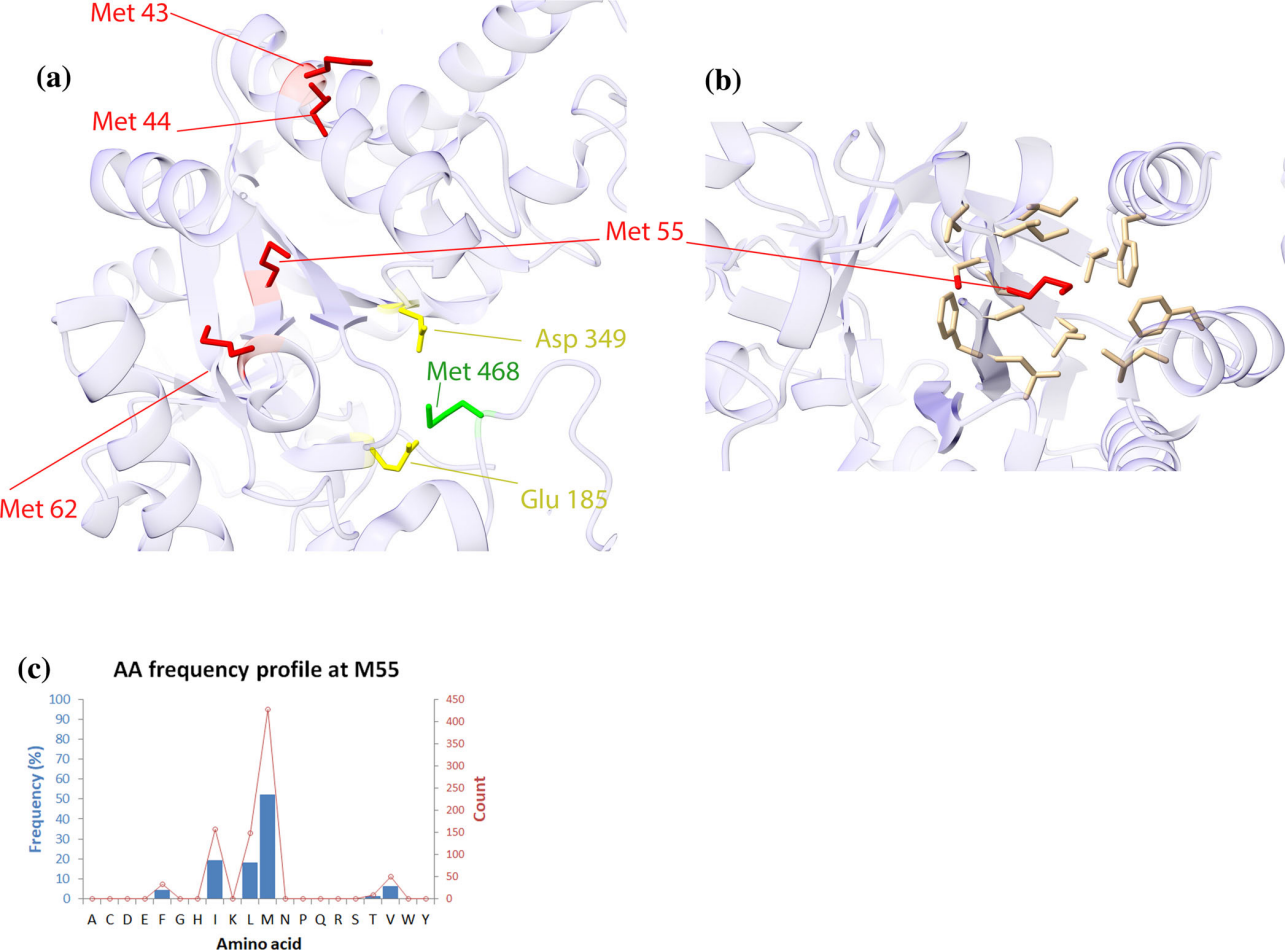
Structural and evolutionary characterization of methionine residues in α‐amylase AmyC (PDB: 2B5D). (a) Three‐dimensional structure of *α*‐amylase AmyC showing experimentally investigated methionine residues Met43, Met44, Met55, and Met62 in red. The catalytic residues Asp349 and Glu185 are shown in yellow, and Met468 is highlighted in green. (b) Local structural environment of Met55 (red) with neighboring residues shown in yellow, illustrating a predominantly hydrophobic local environment. (c) Evolutionary residue‐frequency distribution at position 55, showing partial conservation of methionine (52%) and enrichment of hydrophobic residues including isoleucine (19%), leucine (18%), and valine (6%), consistent with structural constraints at this position.

Structural analysis further shows that Met55 is embedded within a predominantly hydrophobic local environment formed by neighboring residues (Figure 4b). This structural context suggests that oxidative modification at this position may disrupt local packing interactions, thereby affecting enzyme stability.

Evolutionary residue‐frequency analysis provides additional insight into substitution tolerance at this site. The full residue‐frequency distribution derived from the HSSP alignment is provided in Supplementary Table S4. Position 55 shows partial conservation of methionine (~52%), while several hydrophobic residues, including isoleucine (~19%), leucine (~18%), and valine (~6%), are also observed in the evolutionary profile (Figure 4c). The prevalence of hydrophobic residues indicates that maintenance of a nonpolar side chain is structurally favored at this position.

In contrast, Met43 and Met44 display broader evolutionary substitution patterns that include polar and charged residues, suggesting that these sites are subject to weaker structural constraints. Met62 exhibits a more restricted pattern but still shows lower predicted oxidation risk than Met55.

Substitution risk profiling across the amino‐acid alphabet further indicates that isoleucine is predicted to provide the greatest reduction in oxidation susceptibility at position 55 while maintaining structural compatibility with the local environment. This prediction differs from the experimentally tested alanine substitution but highlights an alternative design strategy in which hydrophobic replacements preserve the packing interactions surrounding the hotspot.

Taken together, these results identify Met55 as the primary oxidation‐sensitive site among the experimentally examined residues in *α*‐amylase AmyC and illustrate how EvoMut can refine mutation strategies by integrating oxidation risk with evolutionary substitution constraints.

### 2.9 Case study III: Versatile peroxidase (VP)

To further evaluate the ability of EvoMut to interpret experimentally characterized oxidation‐sensitive systems, we analyzed the versatile peroxidase (VP) engineering dataset reported by Sáez‐Jiménez et al. (2015). In that study, several methionine residues located near the heme cofactor and catalytic environment were investigated through site‐directed mutagenesis to determine their contribution to oxidative inactivation.

EvoMut analysis of the VP structure revealed multiple residues with elevated susceptibility to oxidative modification. Notably, the four methionine residues that were experimentally targeted in the original study (M152, M247, M262, and M265) were all identified among the oxidation‐prone positions in the EvoMut analysis, enabling a direct comparison between computational predictions and experimental observations.

Within this subset, EvoMut assigned ORI values of 0.63 for M247, 0.61 for M265, 0.61 for M152, and 0.57 for M262. These values fall within a relatively narrow range, indicating that oxidative susceptibility in VP is distributed across several methionine residues rather than being dominated by a single critical hotspot.

This pattern is consistent with the experimental findings, where individual substitutions produced only moderate improvements in oxidative stability. No single mutation was sufficient to substantially enhance resistance to oxidative inactivation, suggesting that multiple residues contribute collectively to the observed sensitivity.

From an engineering perspective, this result illustrates an important design principle. When predicted oxidation risk is distributed across several residues with comparable ORI values, mutation of a single site is unlikely to produce a large stabilization effect. Instead, effective stabilization strategies may require coordinated modification of multiple oxidation‐prone residues within the catalytic environment.

Taken together, these findings demonstrate that EvoMut can capture both types of oxidative vulnerability patterns: systems dominated by a single critical hotspot, as observed in A1AT and AmyA, and systems in which susceptibility is distributed across several residues, as observed in versatile peroxidase. This capability is particularly valuable for guiding rational engineering strategies in proteins where oxidative sensitivity arises from multiple contributing sites.

### 2.10 Comparison with existing computational approaches

To place EvoMut in the context of existing tools, we compare it against the current landscape of computational approaches for oxidation site prediction (Supplementary Table S5). Structure‐based classifiers have been developed for cysteine. COPA (Sanchez et al. (2008)) uses inter‐thiol distance, SASA, and calculated pKa to achieve ~80% classification accuracy but requires a solved three‐dimensional structure and incorporates no evolutionary information, and the RAM framework Mapes Jr et al. (2019) extends this by incorporating position‐specific scoring matrices (PSSM) derived from local sequence alignments, reaching AUC = 0.85 for cysteine reactivity prediction. For methionine oxidation, machine learning models by Aledo et al. (2017) and Veredas et al. (2020) achieve AUC 0.81–0.85 using structural and proteomic features; a deep learning approach (MetODeep, (Lopez‐Garcia et al., 2019)) operates directly on sequence without requiring structural input. For therapeutic antibodies, Tavella et al. (2022) demonstrated that incorporating polar neighbor effects via the WCN‐OH descriptor improves sensitivity for partially buried CDR methionines (sensitivity 0.88), though this approach requires computationally intensive molecular dynamics simulations and is restricted to the antibody variable region. A recent structural survey concluded that computational approaches for oxidized cysteine prediction remain underdeveloped, identifying the absence of evolutionary context as a critical gap (Garrido Ruiz et al., 2022). Three limitations are common to all existing methods: restriction to a single residue type, absence of whole‐MSA evolutionary depth, and lack of substitution feasibility guidance. EvoMut addresses all three limitations within a single framework.

## 3 DISCUSSION

Oxidative modification represents a major source of functional instability in many proteins, yet the relationship between chemical susceptibility and practical engineering targets is often complex. The analysis presented here demonstrates that residues with high intrinsic oxidation susceptibility do not necessarily represent the most effective mutation targets. Instead, the functional impact of oxidative modification depends strongly on structural context and evolutionary constraints.

Across the systems examined in this study, EvoMut consistently identified a limited subset of residues that dominate oxidative vulnerability. In A1AT, oxidation risk was concentrated at Met351 and Met358, both located within the reactive center loop (Figure 2). These residues displayed markedly elevated oxidation risk compared with other methionines in the protein (Table 1), consistent with experimental studies demonstrating that oxidation at either site disrupts inhibitory activity. Notably, the single free cysteine of A1AT (Cys232) is intrinsically the most reactive residue in the protein (pK_a_ 6.86; (Griffiths & Cooney, 2002)), yet EvoMut ranks it below the reactive‐center‐loop methionines Met351 and Met358 because oxidation of the loop methionines, not of Cys232, is what abolishes anti‐elastase activity (Taggart et al., 2000), whereas covalent modification of Cys232 leaves inhibitory activity intact (Miyamoto et al., 2000). This illustrates the framework's design: it integrates intrinsic reactivity with functional context rather than ranking residues by reactivity alone. At the same time, evolutionary analysis revealed substantial differences in substitution tolerance between the two sites. Position 351 exhibits a highly restricted evolutionary profile, whereas position 358 shows a broader substitution spectrum. This contrast illustrates that residues with similar oxidative sensitivity may differ significantly in their mutational flexibility.

A similar pattern was observed in the industrial α‐amylase from Alkalimonas amylolytica. EvoMut ranked Met349 as the dominant oxidation‐sensitive site, in agreement with experimental mutagenesis studies in which substitution at this position produced the largest improvement in oxidative stability. Notably, this residue is structurally buried, whereas other methionines with greater solvent accessibility exhibited lower predicted oxidation risk. This observation highlights a key limitation of heuristic approaches that rely primarily on solvent accessibility when identifying oxidation‐sensitive residues. Structural context and functional proximity can outweigh solvent accessibility in determining the functional consequences of oxidative modification.

The analysis of the thermophilic *α*‐amylase AmyC further illustrates how evolutionary information can refine mutation strategies. Experimental mutagenesis identified Met55 as the most influential oxidation‐sensitive position, and substitution with alanine improved oxidative stability. However, the evolutionary profile of this site shows that hydrophobic residues with larger side chains, such as isoleucine, leucine, and valine, are frequently observed at this position. This pattern suggests that substitutions preserving both hydrophobic character and side‐chain volume may better maintain local packing interactions than smaller residues such as alanine (Figure 4). Integrating evolutionary substitution patterns with structural context provides additional guidance for selecting alternative mutation strategies beyond those initially explored experimentally.

In contrast to the previous examples, analysis of versatile peroxidase revealed a distributed pattern of oxidative vulnerability. Several methionine residues exhibited comparable oxidation risk values, and experimental mutagenesis confirmed that individual substitutions produced only moderate improvements in oxidative stability. This pattern suggests that oxidative sensitivity in such systems arises from the combined contribution of multiple residues rather than a single dominant hotspot. In these cases, effective stabilization strategies may require coordinated modification of several oxidation‐prone residues within the catalytic environment. Experimental evidence supports the view that cumulative oxidative modification at multiple residues can produce functional impairments greater than those attributable to any single site (Fuentes‐Lemus et al., 2018; Leinisch et al., 2017; Ozturk et al., 2013).

Taken together, these results highlight two general principles that govern oxidation‐driven protein instability and guide rational engineering strategies. First, oxidation‐sensitive hotspots emerge from the combined influence of chemical susceptibility, structural context, and functional coupling rather than from residue chemistry alone. Second, evolutionary substitution patterns provide a powerful constraint for identifying mutations that are more likely to preserve structural integrity and activity.

By integrating these complementary sources of information, EvoMut enables residue‐level interpretation of oxidative vulnerability while simultaneously restricting the mutational search space to biologically plausible substitutions. This approach allows protein engineers to prioritize a small number of experimentally testable variants, reducing the need for extensive screening while maintaining mechanistic interpretability in protein engineering workflows.

The present study relies on retrospective validation against experimentally characterized systems, which establishes consistency with known outcomes but does not constitute prospective testing. EvoMut also generates novel, residue‐specific predictions, such as the prioritization of isoleucine over the experimentally tested alanine at Met55 in *α*‐amylase AmyC, based on evolutionary substitution patterns and local structural context, that can be directly evaluated by site‐directed mutagenesis. Prospective experimental testing of such predictions represents a natural and important direction for future work.

The current scope of EvoMut is focused on modifications driven by reactive oxygen species, which represent the primary mechanism of residue‐level chemical instability in most protein engineering and biopharmaceutical contexts. However, lysine, arginine, and histidine residues are also susceptible to non‐oxidative electrophilic modifications, including glycation by carbonyl‐containing reducing sugars, carbamylation by cyanate, and quinone adduction. These electrophilic modifications are mechanistically distinct from ROS‐mediated oxidation and share only partial overlap in target residues. Extending EvoMut to encompass electrophilic stress pathways relevant to manufacturing, storage, and pathological in vivo conditions represents an important direction for future development.

## 4 CONCLUSION

Oxidative modification remains a major challenge in the stability and long‐term functionality of many proteins used in biomedical and industrial applications. The analyses presented here demonstrate that oxidative vulnerability cannot be reliably predicted from residue chemistry or solvent accessibility alone. Instead, susceptibility emerges from the combined influence of structural context, functional proximity, and evolutionary constraints.

EvoMut provides an interpretable residue‐level framework that separates the identification of oxidation‐sensitive hotspots from the evaluation of mutation feasibility. By integrating structural features with evolutionary substitution patterns, the framework enables prioritization of mutation strategies that are both chemically meaningful and biologically plausible.

Application of EvoMut to experimentally characterized systems illustrates how this integrative approach can distinguish dominant oxidative hotspots from structurally constrained residues and clarify when single‐site or multi‐site engineering strategies may be required. Together, these capabilities support design of oxidation‐resistant proteins while reducing the experimental search space in protein engineering projects.

## 5 METHODS

### 5.1 Implementation

EvoMut is implemented as a web‐accessible analytical framework written in Python and designed with a modular architecture to enable flexible residue‐level analysis. The implementation separates data ingestion, structural analysis, evolutionary annotation, risk scoring, and mutation assessment into independent computational modules, allowing each stage of the workflow to be evaluated or extended without affecting the overall framework.

Structural features are extracted directly from the input protein structure using established bioinformatics libraries for parsing coordinate files and analyzing residue‐level properties. Solvent accessibility, secondary structure, and inter‐residue contacts are computed from the three‐dimensional coordinates. Evolutionary information is incorporated through residue‐specific profiles derived from multiple sequence alignments.

All intermediate calculations are handled in memory during runtime to ensure session isolation and reproducibility. The framework is deployed as an interactive web application that integrates numerical analysis with synchronized sequence‐level and structure‐level visualization, allowing users to explore oxidation risk and mutation feasibility in an iterative manner.

### 5.2 Input data

#### 5.2.1 Protein structure

EvoMut requires a three‐dimensional protein structure obtained from the Protein Data Bank (PDB). Users may provide a PDB identifier or upload a local coordinate file. For each structure, EvoMut automatically detects available chains and extracts the amino‐acid sequence directly from atomic coordinate records, ensuring consistency between structural and sequence representations. EvoMut automatically compares the amino‐acid sequence extracted from the PDB coordinate file against the canonical UniProt sequence (Dana et al., 2019) to detect engineered mutations, truncations, or crystallographic substitutions that may alter oxidation risk scoring. When a discrepancy is detected at an oxidation‐sensitive residue, for example, a cysteine‐to‐alanine substitution introduced to prevent disulfide bond formation in a crystal structure, EvoMut prompts the user to restore the wild‐type residue. Restored side‐chain coordinates are rebuilt using a NeRF‐based algorithm (Parsons et al., 2005) with modal chi angles from the Dunbrack rotamer library (Shapovalov & Dunbrack Jr., 2011) and standard Engh and Huber bond geometry (Engh & Huber, 1991).

Residue‐level structural features are derived from the structure, including relative solvent accessibility, secondary structure assignment, and spatial proximity between residues. These features are subsequently used to evaluate residue exposure, identify structurally relevant regions, and determine neighboring residues within a user‐defined distance threshold. Solvent accessibility contributes to the oxidation risk score as a continuous factor rather than a binary filter. By default, the surface exposure threshold is set to zero, meaning that all residues (including those in buried positions) are evaluated for oxidation susceptibility. This design accommodates oxidants that are not strictly limited by surface exposure, such as singlet oxygen and ultraviolet radiation. The threshold can be adjusted interactively through the EvoMut interface to focus analysis on surface‐exposed residues when the experimental context warrants it.

#### 5.2.2 Evolutionary profile

Evolutionary information is incorporated through residue‐specific sequence profiles derived from multiple sequence alignments. EvoMut automatically retrieves HSSP‐formatted multiple sequence alignment profiles from a local instantiation of the XSSP database (Schneider et al., 1997) using the protein identifier derived from the PDB structure, eliminating the need for manual profile submission or upload.

These profiles provide position‐specific amino‐acid frequencies and conservation metrics that reflect evolutionary constraints at each residue. Within EvoMut, evolutionary data serve two complementary purposes: they contribute modestly to oxidation risk estimation by penalizing highly conserved positions and they guide the identification of plausible amino‐acid substitutions based on observed evolutionary variability.

Optional annotations, such as known functional residues or sequence features, may also be incorporated when available in UniProt annotations to refine site‐context evaluation, although they are not required for analysis.

The reliability of the evolutionary component depends on the depth of the multiple sequence alignment. Profiles containing fewer than 30 aligned sequences are considered insufficient for reliable evolutionary scoring; EvoMut automatically issues a blocking warning in this case. Profiles with 30–49 sequences trigger a caution message, and 50 or more sequences are considered adequate. For proteins with limited evolutionary coverage, users are advised to reduce or disable the conservation weight (*w*_K_) and to interpret position‐specific substitution frequencies with caution.

### 5.3 Oxidation risk assessment

For each residue, EvoMut estimates oxidation susceptibility by integrating structural, chemical, and evolutionary determinants into a unified scoring framework. The ORI is defined as a weighted combination of multiple normalized factors:

$$\mathrm{ORI}=w_{E}\cdot E+w_{C}\cdot C+w_{K}\cdot \left(1-K\right)+w_{S}\cdot S$$

where *E* represents relative solvent accessibility (RSA), *C* represents intrinsic chemical susceptibility of the amino acid, *K* represents the evolutionary frequency of the residue in the multiple sequence alignment, *S* represents the functional‐site context score, reflecting proximity to annotated catalytic or binding residues.

The coefficients *w*_*E*_, *w*_*C*_, *w*_*K*_, and *w*_*S*_ define the relative contribution of each factor. All variables are normalized to the range between 0 and 1, resulting in a continuous per‐residue ORI value between 0 (low oxidation susceptibility) and 1 (high susceptibility).

EvoMut treats cysteine, methionine, tryptophan, and tyrosine as primary oxidation targets, reflecting their consistently high intrinsic chemical susceptibility across the major oxidant species. The cysteine weight refers to a defined exemplar, a solvent‐exposed free thiol with an unperturbed pK_a_ (about 8.3) at pH 7.4; disulfide‐bonded cysteines are detected from the structure and scored separately. Histidine, lysine, and arginine are incorporated as secondary targets, with lower default chemical susceptibility weights in the screening profile (*C* = 0.45, 0.08, and 0.04, respectively) because they are efficiently oxidized only under specific conditions (histidine primarily through metal‐catalyzed oxidation; lysine and arginine through metal‐catalyzed carbonylation and non‐oxidative electrophilic pathways). All chemical susceptibility weights are adjustable by the user through the EvoMut interface.

Default weighting parameters are provided in Supplementary Tables S1 and S2 and can be interactively adjusted through the EvoMut interface. The chemical susceptibility component of the ORI (term C) assigns residue‐specific weights derived by log‐linear normalization of published second‐order rate constants (pH 7.4, 25°C) and, where reliable absolute rate constants are unavailable, of documented experimental reactivity hierarchies, normalized separately within each oxidant class (Dorta et al., 2019; Folzer et al., 2015; Pattison et al., 2007; Sjöberg et al., 2018). EvoMut defines six single oxidant species rather than combined categories: hydrogen peroxide (H_2_O_2_), peroxyl radicals (ROO•), hypochlorous acid (HOCl), metal‐catalyzed oxidation (MCO, generating •OH), singlet oxygen (^1^O_2_), and UV photo‐oxidation. The species can be selected singly or in combination, and no two species with different reaction chemistry are merged. When the oxidant is unspecified, the default screening profile is computed as the equal‐prior geometric mean of the six single‐species weights (renormalized to a maximum of 1.00) rather than assigned by hand; as a result, a solvent‐exposed free cysteine heads the ranking: Cys (1.00) >Met (0.74) >Trp (0.50) >His (0.45) >Tyr (0.37) >Lys (0.08) >Arg (0.04). For HOCl, cysteine and methionine are the primary direct targets (rate constants 3.6 × 10^8^ and 3.8 × 10^7^ M^−1^ s^−1^, respectively, the cysteine value revised upward in later measurements (Pattison & Davies, 2001; Storkey et al., 2014)); secondary chloramine‐transfer reactions extend reactivity both to tryptophan and tyrosine (Pattison et al., 2007) and, with even larger rate constants, to cysteine and methionine themselves (Peskin & Winterbourn, 2001). Metal‐catalyzed oxidation preferentially carbonylates histidine, lysine, and arginine at Fenton‐active sites (Amici et al., 1989; Stadtman, 1990), and singlet oxygen targets histidine, tryptophan, and methionine on the basis of reactive quenching rate constants (Matheson et al., 1975; Michaeli & Feitelson, 1994). Users may also define custom weights. The quantitative basis and full weight tables for each species are provided in Supplementary Methods 4 (Supplementary Table S1; supporting rate constants and reactivity data, Supplementary Tables S6–S9).

Importantly, because different oxidants exhibit markedly different diffusion and penetration capacities, each species also reconfigures the four ORI term weights according to three oxidant penetration tiers (Supplementary Table S10). HOCl, a large charged species, cannot penetrate the protein interior, so its profile elevates the solvent‐exposure weight (*w*_*E*_ = 0.15) relative to the screening default (*w*_*E*_ = 0.07). Conversely, UV light penetrates all regions of a protein structure irrespective of surface exposure, and singlet oxygen has a short lifetime (~3 μs in aqueous solution) but diffuses ~200 nm from its point of generation, giving it access to buried residues; the singlet‐oxygen and UV profiles therefore minimize the RSA contribution (*w*_*E*_ = 0.05) and elevate the chemistry weight (*w*_*C*_ = 0.55). H_2_O_2_, a small neutral molecule, diffuses through protein channels (Dorta et al., 2019; Sjöberg et al., 2018), and its profile similarly reduces *w*_*E*_ (0.05) while emphasizing chemistry selectivity (*w*_*C*_ = 0.45). For metal‐catalyzed oxidation, hydroxyl radicals are generated at metal‐binding sites and react within a diffusion radius of ~1–2 nm (Stadtman, 1990); proximity to functional/metal‐binding sites is therefore the dominant factor, and the MCO profile elevates the site‐context weight (*w*_*S*_ = 0.50). The complete set of per‐species ORI term weights is provided in Supplementary Table S11. Users who select a species retain full control over individual slider adjustments.

### 5.4 Mutation feasibility assessment

Residues identified as high‐risk oxidation hotspots are subjected to a second‐stage analysis focused on mutation feasibility. At this stage, evolutionary information plays a central role. Position‐specific substitution patterns derived from multiple sequence alignments are used to determine which amino‐acid replacements are evolutionarily tolerated at each site. Candidate substitutions are therefore restricted to residues that occur within the observed evolutionary substitution spectrum.

Substitutions are subsequently evaluated based on their reduced chemical susceptibility relative to the native residue while maintaining compatibility with evolutionary constraints. This staged approach ensures that chemically favorable substitutions are also consistent with the structural and functional constraints reflected in the evolutionary record.

### 5.5 Analysis of local structural context

Protein stability and function often arise from networks of interactions among neighboring residues rather than from the behavior of a single residue in isolation. EvoMut therefore evaluates residues that are spatially adjacent to each identified hotspot. Structural contact analysis is used to identify neighboring residues within a defined spatial cutoff. Evolutionary patterns of these surrounding residues are examined to assess whether limited adjustments within the local environment may improve compatibility with the proposed substitution.

This analysis supports context‐aware mutation strategies in which small adjustments within local interaction networks may mitigate destabilizing effects or preserve catalytic functionality.

### 5.6 Output and visualization

EvoMut produces residue‐level outputs that are fully traceable and interpretable. These include oxidation risk scores for all residues in the protein, identification of high‐risk oxidation hotspots, evolutionarily supported substitution candidates for each hotspot, and contextual information about neighboring residues and local structural contacts.

Results are presented through an interactive interface that integrates tabular summaries, sequence‐level annotation, and three‐dimensional structural visualization. These synchronized views allow users to examine oxidation risk and mutation feasibility within their structural and evolutionary context. All output tables can be exported for downstream analysis or experimental planning. Screenshots illustrating the EvoMut workflow and analysis interface are provided in Supplementary Figures S1–S3.

## AUTHOR CONTRIBUTIONS

**Seyed Shahriar Arab:** Conceptualization; methodology; software; formal analysis; visualization; investigation; writing – original draft; writing – review and editing; data curation. **Chenlin Hsieh:** Validation; writing – review and editing; writing – original draft. **Nathan E. Lewis:** Resources; writing – review and editing; conceptualization; supervision; funding acquisition.

## Supporting information


**Supplementary Figure S1:** EvoMut web interface—Step 1: Input configuration. Users provide the PDB structure, HSSP alignment, and chain identifier used for the analysis. Optional parameters allow specification of reference sequence and functional‐site residues when required.
**Supplementary Figure S2:** EvoMut web interface—Step 2: Structural analysis. The program evaluates residue‐level structural properties including solvent accessibility, spatial neighborhood, and proximity to functional or interface residues.
**Supplementary Figure S3:** EvoMut web interface—Step 3: Oxidation risk and mutation analysis. The EvoMut framework calculates oxidation risk scores and identifies candidate substitutions based on evolutionary substitution profiles.
**Supplementary Table S1:** Chemical susceptibility weights (C) for oxidation‐prone residues across the six single EvoMut oxidant species and the computed screening (aggregate) default.
**Supplementary Table S2:** Default weighting coefficients used in the ORI scoring function.
**Supplementary Table S3:** Complete oxidation risk index (ORI) values for oxidation‐prone residues in human *α*_1_‐antitrypsin.
**Supplementary Table S4:** Evolutionary residue‐frequency distribution for Met55 in *α*‐amylase AmyC derived from the HSSP alignment.
**Supplementary Table S5:** Comparison of computational approaches for oxidation site prediction in proteinsSupplementary Table S6 Chemical susceptibility weights and supporting rate data for hydrogen peroxide and peroxyl radicals (ROO•), listed separately.
**Supplementary Table S7:** Chemical susceptibility weights and HOCl second‐order rate constants for hypochlorous acid (HOCl).
**Supplementary Table S8:** Chemical susceptibility weights and target roles for metal‐catalyzed oxidation (MCO / •OH).
**Supplementary Table S9:** Chemical susceptibility weights for singlet oxygen (^1^O₂) and UV photo‐oxidation, listed separately.
**Supplementary Table S10:** Oxidant penetration tiers used to assign the solvent‐exposure weight (*w*_E_).
**Supplementary Table S11:** Per‐species ORI term weights (*w*_E_, *w*_C_, *w*_S_, *w*_K_) and their derivation basis, including the computed screening (aggregate) default used when the oxidant is unspecified.

## Data Availability

The EvoMut framework is available as a web server at https://proteus.cmm.uga.edu/evomut. The datasets supporting this study are publicly available in Zenodo at https://zenodo.org/records/19052250. Additional methodological details and supporting material are provided in the Supplementary Information.
