# Supplementary material for: EvoMut: A computational framework for engineering oxidative stability in proteins
Source: Protein Sci. 2026 Sep 15;35(10):e70774. doi: 10.1002/pro.70774 (PMC13575976; doi:10.1002/pro.70774)
Supplement: Supplementary file 1 [file PRO-35-e70774-s001.docx]

Supplementary Information

**EvoMut: A Computational Framework for Engineering Oxidative Stability in Proteins**

Contents

[**Supplementary Methods**	3](#_Toc236151875)

[Sup. Method 1. Definition of the Oxidation Risk Index (ORI)	3](#_Toc236151876)

[Sup. Method 2. Evolutionary substitution analysis and mutation feasibility	4](#_Toc236151877)

[Sup. Method 3. Identification of functional and interface residues	4](#_Toc236151878)

[Sup. Method 4. Quantitative basis for oxidant-specific chemical susceptibility weights	4](#_Toc236151879)

[**Supplementary Figures**	10](#_Toc236151880)

[Sup. Fig. 1: Step 1 (Input data)	10](#_Toc236151881)

[Sup. Fig. 2: Step 2 (Parameters setting)	11](#_Toc236151882)

[Sup. Fig. 3: Step 3 (Analysis data and building constructions)	12](#_Toc236151883)

[**Supplementary Tables**	13](#_Toc236151884)

[Supplementary Table S1. Default Chemical susceptibility weights	13](#_Toc236151885)

[Supplementary Table S2. Default weighting coefficients	13](#_Toc236151886)

[Supplementary Table S3. Oxidation-prone candidates’ info	13](#_Toc236151887)

[Supplementary Table S4. Evolutionary residue-frequency distribution	15](#_Toc236151888)

[Supplementary Table S5. Comparison of computational approaches	16](#_Toc236151889)

[Supplementary Table S6. Chemical susceptibility weights for hydrogen peroxide and peroxyl radicals (ROO•), listed separately	16](#_Toc236151890)

[Supplementary Table S7. Chemical susceptibility weights for hypochlorous acid (HOCl)	17](#_Toc236151891)

[Supplementary Table S8. Chemical susceptibility weights for metal-catalyzed oxidation (MCO / •OH)	17](#_Toc236151892)

[Supplementary Table S9. Chemical susceptibility weights for singlet oxygen (¹O₂) and UV photo-oxidation, listed separately	17](#_Toc236151893)

[Supplementary Table S10. Oxidant penetration tiers	17](#_Toc236151894)

[Supplementary Table S11. Per-species ORI term weights and redistribution	18](#_Toc236151895)

[**Supplementary Data Availability**	18](#_Toc236151896)

[**References:**	18](#_Toc236151897)

# **Supplementary Methods**

## Sup. Method 1. Definition of the Oxidation Risk Index (ORI)

The oxidation risk index (ORI) used in EvoMut quantifies residue-level susceptibility to oxidative modification as a weighted combination of multiple independent determinants. The ORI score is defined as:

ORI = *w_E_*⋅E + *w_C_*⋅C + *w_K_*⋅(1-K) + *w_S_*⋅S

where:

**E (Exposure)** Relative solvent accessibility (RSA) derived from structural analysis.

**C (Chemical liability)** Residue-type–specific intrinsic susceptibility to oxidative modification. Seven residue types are assigned non-zero susceptibility scores: methionine (M, 1.00), cysteine (C, 0.90), tryptophan (W, 0.60), tyrosine (Y, 0.50), histidine (H, 0.45), lysine (K, 0.25), and arginine (R, 0.20). Histidine, lysine, and arginine undergo oxidation primarily under metal-catalyzed and carbonylation conditions[1]. All other residue types receive a score of zero. Full values are provided in Supplementary Table S1.

**S (Site context)** Proximity of the residue to annotated functional or binding sites derived from UniProt annotations or structural interface analysis.

**K (Conservation)** Normalized evolutionary conservation score derived from multiple sequence alignment entropy.

The coefficients w_E_, w_C_, w_K_, and w_S_ define the relative contribution of each factor. All coefficients are scaled to the range**[0,1]** prior to integration. Default weighting coefficients used throughout this study are summarized in Supplementary Table S2.

These values were selected to reflect the dominant contributions of intrinsic chemical susceptibility and structural context while allowing evolutionary conservation to modulate, but not dominate, oxidation risk estimation.

## Sup. Method 2. Evolutionary substitution analysis and mutation feasibility

Evolutionary substitution profiles were derived from [XSSP/HSSP multiple sequence alignments](https://www3.cmbi.umcn.nl/xssp/), which provide residue-specific amino acid frequencies across homologous protein sequences.

For each residue position, amino acid frequencies were extracted and normalized to represent the observed evolutionary tolerance of substitutions at that site.

Mutation feasibility was evaluated only after identification of residues with elevated oxidation risk and was treated as a separate analytical stage. Candidate substitutions were ranked based on:

- Observed evolutionary frequency at the target position
- Structural compatibility inferred from the local residue environment (e.g., surface-exposed vs buried residues)
- Absence of known pathogenic annotations when available (e.g., ClinVar annotations for human proteins)

This staged design ensures that oxidation susceptibility and mutation feasibility are evaluated independently, preventing chemically reactive residues from being automatically interpreted as suitable mutation targets.

## Sup. Method 3. Identification of functional and interface residues

Functional context contributes to oxidation risk estimation through the site context component (S) of the ORI score.

For multimeric protein structures, EvoMut automatically identifies residues that participate in inter-chain contacts. Residues located within a defined distance threshold from atoms belonging to another chain are classified as interface residues and contribute to the functional-site context score.

For single-chain proteins, EvoMut can optionally incorporate manually specified functional residues such as catalytic or binding residues when available. These annotations may be derived from UniProt functional features or experimental literature.

This flexible design allows EvoMut to integrate both automatically detected structural interfaces and manually annotated functional sites to refine residue-level context evaluation.

## Sup. Method 4. Quantitative basis for oxidant-specific chemical susceptibility weights

**4.1 Overview**

The Oxidation Risk Index (ORI) includes a chemical susceptibility term (Cᵢ) that weights each residue according to its intrinsic reactivity toward reactive oxygen and related species. EvoMut defines six individual oxidant species: hydrogen peroxide (H₂O₂), peroxyl radicals (ROO•), hypochlorous acid (HOCl), metal-catalyzed oxidation (MCO, generating •OH), singlet oxygen (¹O₂), and ultraviolet photo-oxidation (UV). Each species is parameterized independently, and the species can be selected singly or in any combination (Section 4.8). No two species with distinct reaction chemistry are merged into a fixed composite category.

Within each species, the chemical susceptibility weights are derived by log-linear normalization of published second-order rate constants at pH 7.4 and 25 °C, Cᵢ = (log₁₀kᵢ − log₁₀kmin) / (log₁₀kmax − log₁₀kmin), normalized separately within each oxidant class so that the most reactive residue of that class scores 1.00. Reliable absolute rate constants are not available for every species; where they are not, the weights are derived from an alternative, explicitly stated experimental basis. Each species therefore carries a basis label: log-k (H₂O₂, HOCl, ¹O₂), consumption-rank (ROO•), carbonyl-yield (MCO), or chromophore-absorption (UV). Stating the basis per species prevents rate constants of different provenance from being compared as if they were on one scale.

The default screening profile used when the oxidant is unspecified is not hand-assigned; it is computed from the six single-species profiles by an equal-prior aggregation rule (Section 4.8).

**4.2 Hydrogen peroxide (H₂O₂)**

Hydrogen peroxide reacts via a two-step nucleophilic mechanism with thiolate anions. The rate constant for free Cys thiolate + H2O2 is approximately 17 M⁻¹s⁻¹ at pH 7.4[2,3]. For Met, direct oxidation by H2O2 is substantially slower (k ≈ 0.08–0.10 M⁻¹s⁻¹ for free Met)[4,5], but Met sulfoxide formation is the dominant degradation pathway in biopharmaceuticals under H2O2 stress conditions owing to the abundance of surface-exposed Met residues. Trp, Tyr, His, Lys, and Arg do not undergo meaningful direct oxidation by H2O2.

Accordingly, this species assigns Cys the maximum weight (1.00) and Met a substantially lower weight (0.47), with all other residues at 0.00. This corrects an inversion in the previous merged preset, where Met (1.00) outscored Cys (0.95) even though the measured thiolate rate constant is about 170-fold larger than that of Met; separating H₂O₂ from peroxyl radicals removes that artifact. The H₂O₂ weights and rate data are listed in Supplementary Table S6.

**4.3 Peroxyl radicals (ROO•)**

Peroxyl radicals (generated experimentally by AAPH thermolysis) show a selectivity distinct from H₂O₂: Met and Cys are the most consumed residues, followed by Trp and Tyr, with His moderately reactive and Lys/Arg essentially inert [6–9]. Reliable absolute second-order rate constants for ROO• with individual protein side chains are sparse and protein-context-dependent; the weights for this species are therefore derived from the experimental consumption hierarchy rather than from log-k, and are labeled accordingly (basis: consumption-rank).

The resulting weights are Cys 1.00, Met 0.90, Trp 0.65, Tyr 0.55, His 0.30, Lys 0.10, and Arg 0.10. In particular, His is assigned a non-negligible weight (0.30) rather than a near-zero value, which implements Reviewer 1’s Comment 3. The limited kinetic data for His oxidation by peroxyl radicals reflect the large number of products formed and the resulting difficulty of quantification, and should not be read as evidence of low reactivity; the former near-zero His value was an artifact of averaging with H₂O₂, which does not react with His.

These weights and the supporting rate data are provided in Supplementary Table S6.

**4.4 Hypochlorous acid (HOCl)**

Pattison & Davies determined absolute second-order rate constants for reactions of HOCl with protein side chains at pH 7.4 [10], providing the quantitative basis for this species (basis: log-k). Weights were derived by log₁₀ normalization of these rate constants. Storkey et al. revised the Cys + HOCl rate constant upward to 3.6×10⁸ M⁻¹s⁻¹ [11], placing Cys and Met as the two primary targets (Cys 1.00, Met 0.86).

An upward correction is applied to Trp and Tyr to account for secondary chloramine-transfer reactions: His and Lys side-chain chloramines transfer oxidizing equivalents to Trp and Tyr in intact proteins, making these residues more susceptible than their direct rate constants suggest [12,13].

In addition, His- and Lys-derived chloramines also transfer oxidizing equivalents to the sulfur-containing residues Cys and Met, so that a fraction of the total HOCl dose reaches these residues by a secondary, longer-range route in addition to direct reaction [14]. Because Cys and Met are already at or near the maximum of this species, this secondary pathway reinforces their top ranking rather than changing it; it is noted here for completeness and because it explains the persistence of Cys/Met oxidation at sites remote from the initial HOCl reaction. Arg receives the lowest weight (0.00), as both its direct rate constant and its secondary modification potential are the lowest of the seven residues. These weights and rate constants are listed in Supplementary Table S7.

**4.5 Metal-catalyzed oxidation (MCO / •OH)**

Metal-catalyzed oxidation involves site-specific Fenton-type reactions at metal-binding residues, generating hydroxyl-radical equivalents that attack nearby side chains. Stadtman established that MCO systems specifically convert His, Arg, Lys and Pro side chains to carbonyl derivatives, while Met is oxidized to sulfoxide and Cys to mixed disulfide; Trp and Tyr are not primary carbonyl targets [15]. Amici et al. [1], Schöneich [16] and Requena et al. [17] provide the quantitative carbonyl-yield hierarchy: His, Arg, Lys > Pro >> Met, Cys.

The rate constant of •OH itself is diffusion-limited (≈10⁹–10¹⁰ M⁻¹s⁻¹) and essentially non-discriminating across all residues, so it cannot be used to rank susceptibility. The weights for this species are therefore derived from the experimental carbonyl-yield hierarchy rather than from k (basis: carbonyl-yield), giving His 1.00, Lys 0.85, Arg 0.80, Cys 0.60, Met 0.35, and Trp = Tyr = 0.20. Making the basis explicit here prevents the mistaken expectation that near-equal •OH rate constants should translate into near-equal weights.

These weights are provided in Supplementary Table S8.

**4.6 Singlet oxygen (¹O₂)**

Matheson et al. measured total ¹O₂ quenching rate constants for amino acids in D₂O: His (17×10⁷ M⁻¹s⁻¹) > Trp (9×10⁷) > Met (3×10⁷) ≫ Ala. Total quenching includes both physical (non-reactive) and chemical (reactive) pathways [18]. Michaeli & Feitelson quantified the reactive fraction: Trp has a substantially higher reactive quenching fraction (~65%) than His (~35%), so the reactive rate constants of His and Trp are nearly equal [19].

Weights are assigned from the reactive rate constants (basis: log-k): His 1.00, Trp 0.95, Met 0.85, Tyr 0.65, Cys 0.60, and Lys = Arg = 0.00. Separating ¹O₂ from UV allows Met and Cys to carry the substantial ¹O₂ reactivity that the kinetic data support, reactivity that was masked when the category was dominated by the UV chromophores Trp and Tyr. This is the direct implementation of Reviewer 1’s Comment 1 for the photochemical classes.

These weights and supporting data are provided in Supplementary Table S9.

**4.7 Ultraviolet photo-oxidation (UV)**

Direct UV photo-oxidation is governed by chromophore absorption near 280 nm[20]. Tryptophan is the dominant absorber and primary photolytic target (1.00), followed by tyrosine (0.45) and histidine (0.25); free methionine and free cysteine have negligible direct absorption and are assigned low weights (Met 0.05, free Cys 0.10). Indirect, ¹O₂-mediated damage to Met and Cys under photosensitizing conditions is captured by the separate ¹O₂ species (Section 4.6) and, when both act together, by co-exposure selection (Section 4.8) [21–23].

Unlike every other species, UV assigns the disulfide-bonded cysteine a higher weight (0.40) than the free thiol (0.10). Disulfide bonds are photolabile: they absorb in the near-UV and undergo homolytic S–S cleavage [24,25], so a structural disulfide is more, not less, vulnerable under UV. This species-dependent disulfide weight is stored per species rather than as a single global constant.

These weights and supporting data are provided in Supplementary Table S9.

**4.8 Unspecified / mixed exposure: computed screening**

When the oxidant environment is unknown or deliberately non-specific (for example, early screening under mixed manufacturing or cellular stress), EvoMut applies an equal-prior aggregate over all six species rather than a separate “general ROS” category. The aggregate chemical weight of each residue is the geometric mean of its six single-species weights (with a small offset ε = 0.01 to accommodate zero entries), renormalized so that the most reactive residue scores 1.00:

C_Screen_(i) = normalize [GM (C_H₂O₂_, C_ROO•_, C_HOCl_, C_MCO_, C_¹O₂_, C_UV_)].

The geometric mean (rather than an arithmetic mean) is used deliberately: a residue that is highly reactive in only one class is not promoted to the top of the default ranking; a residue must be broadly reactive across species to score high. The computed screening weights are listed in Supplementary Table S1.

Free cysteine emerges at the top of the screening ranking as a result of aggregating literature-grounded single-species weights, not as an assigned value. This directly addresses the reviewer’s point that a free, solvent-exposed thiol should head the intrinsic-reactivity ranking. The exemplar for the cysteine weight is defined explicitly as a free, solvent-exposed thiol at pH 7.4 (pKa ≈ 8.3); catalytic or micro-environment-activated low-pKa thiols are captured through the site-context term S rather than through C (Section 4.10), and disulfide-bonded cysteines are scored separately (Section 4.9).

**Selecting more than one species**

The screening default above is one instance of a general rule: the six species are parameterized independently, so the user can select any subset under one of two combination modes.

Co-exposure (species act simultaneously, e.g. an ICH Q1B photostability test combining UV and ¹O₂). Each residue takes the maximum chemical weight over the selected species; the exposure weight w_E_ takes the minimum over the selected species (the most penetrating species dominates access to buried sites); the site-context weight w_S_ takes the maximum.

Unknown oxidant (exactly one species is present, but its identity is unknown). Chemical weights are combined by geometric mean and the ORI term weights by arithmetic mean. Applying this mode to all six species reproduces the screening profile tabulated above and the screening term weights of Section 4.10 (w_E_ = 0.07, w_C_ = 0.44, w_S_ = 0.34, w_K_ = 0.15); this all-species, unknown-oxidant combination is the program default when no species is explicitly selected.

**4.9 Summary: Chemical Susceptibility Weights across all species**

Supplementary Table S1 summarizes the chemical susceptibility weights (Cᵢ) for all six single species, the computed screening (aggregate) column, and the per-species disulfide-cysteine weight. Values range from 0 (no contribution) to 1.00 (maximum susceptibility within that oxidant class). The disulfide-bonded cysteine is scored with a protected weight of 0.05 for every species except UV, where it is 0.40 (Section 4.7); under the aggregate screening profile the disulfide weight follows the same geometric-mean combination as the other weights.

**4.10 Derivation of ORI Term Weights (w_E_, w_C_, w_S_, w_K_) per species**

**4.10.1 Background: the four ORI term weights**

The Oxidation Risk Index is defined as:

ORI = *w_E_*⋅E + *w_C_*⋅C + *w_K_*⋅(1-K) + *w_S_*⋅S

where E is solvent exposure (RSA), C is chemical susceptibility, S is functional/structural site context (active-site or binder-contact proximity), and K is evolutionary conservation. The four weights sum to 1.00.

The derivation principles are unchanged: w_K_ is invariant at 0.15 for all species (sequence conservation is intrinsic to the protein and independent of the external oxidant); w_E_ is set from the oxidant’s penetration capacity via three tiers (high-surface-dependence 0.15, standard 0.10, low-surface-dependence 0.05); freed weight from a lower w_E_ is transferred to w_C_, and MCO is the exception in which w_S_ is elevated because •OH acts within a ~1–2 nm radius of its metal-coordination site [13]. The default weighting coefficients are given in Supplementary Table S2, the penetration tiers in Supplementary Table S10, and the per-species term weights in Supplementary Table S11.

The screening default term weights (w_E_ = 0.07, w_C_ = 0.44, w_S_ = 0.34, w_K_ = 0.15) are the arithmetic mean of the six single-species term weights.

**Supplementary Figures**

## Sup. Fig. 1: Step 1 (Input data)

Supplementary Figure S1 EvoMut web interface – Step 1: Input configuration. Users provide the PDB structure, HSSP alignment, and chain identifier used for the analysis. Optional parameters allow specification of reference sequence and functional-site residues when required.

## Sup. Fig. 2: Step 2 (Parameters setting)

Supplementary Figure S2 EvoMut web interface – Step 2: Structural analysis. The program evaluates residue-level structural properties including solvent accessibility, spatial neighborhood, and proximity to functional or interface residues.

## Sup. Fig. 3: Step 3 (Analysis data and building constructions)

Supplementary Figure S3 EvoMut web interface – Step 3: Oxidation risk and mutation analysis. The EvoMut framework calculates oxidation risk scores and identifies candidate substitutions based on evolutionary substitution profiles.

# **Supplementary Tables**

## Supplementary Table S1. Default Chemical susceptibility weights

Supplementary Table S1 Chemical susceptibility weights (C) for oxidation-prone residues across the six single EvoMut oxidant species and the computed screening (aggregate) default.

| **Residue** | **Code** | **H₂O₂** | **ROO•** | **HOCl** | **MCO** | **¹O₂** | **UV** | **Screening (aggregate)** | **Primary oxidation product(s)** |
| --- | --- | --- | --- | --- | --- | --- | --- | --- | --- |
| Methionine | M | 0.47 | 0.90 | 0.86 | 0.35 | 0.85 | 0.05 | 0.74 | Methionine sulfoxide (MetO); sulfone (MetO₂) |
| Cysteine (free) | C | 1.00 | 1.00 | 1.00 | 0.60 | 0.60 | 0.10 | 1.00 | Sulfenic/sulfinic/sulfonic acid; disulfide |
| Tryptophan | W | 0.00 | 0.65 | 0.55 | 0.20 | 0.95 | 1.00 | 0.50 | N-formylkynurenine, kynurenine, hydroxy-Trp |
| Tyrosine | Y | 0.00 | 0.55 | 0.35 | 0.20 | 0.65 | 0.45 | 0.37 | DOPA, dityrosine, nitrotyrosine |
| Histidine | H | 0.00 | 0.30 | 0.50 | 1.00 | 1.00 | 0.25 | 0.45 | 2-Oxohistidine; Asp/Asn |
| Lysine | K | 0.00 | 0.10 | 0.32 | 0.85 | 0.00 | 0.00 | 0.08 | 2-Aminoadipic semialdehyde (carbonyl) |
| Arginine | R | 0.00 | 0.10 | 0.00 | 0.80 | 0.00 | 0.00 | 0.04 | Glutamic semialdehyde (carbonyl) |
| Cysteine (S–S) | C | 0.05 | 0.05 | 0.05 | 0.05 | 0.05 | 0.40 | 0.07 | Protected disulfide; photolabile under UV |

## Supplementary Table S2. Default weighting coefficients

Supplementary Table S2 Default weighting coefficients used in the ORI scoring function.

| **Parameter** | **Description** | **Default value** |
| --- | --- | --- |
| w_E_ | Solvent exposure weight | 0.07 |
| w_C_ | Chemical susceptibility weight | 0.44 |
| w_S_ | Functional site context weight | 0.34 |
| w_K_ | Evolutionary conservation weight | 0.15 |

## Supplementary Table S3. Oxidation-prone candidates’ info

Supplementary Table S3 Complete oxidation risk index (ORI) values for oxidation-prone residues in human α₁-antitrypsin.

| **pos** | **WT** | **WT frq.** | **RSA** | **Chemistry** | **Func. proximity** | **ORI** |
| --- | --- | --- | --- | --- | --- | --- |
| 358 | M | 26 | 0.88 | 1.0 | 1.00 | 0.95 |
| 351 | M | 3 | 0.56 | 1.0 | 0.83 | 0.89 |
| 226 | M | 21 | 0.13 | 1.0 | 0.63 | 0.73 |
| 281 | R | 25 | 0.44 | 0.2 | 1.00 | 0.63 |
| 282 | R | 52 | 0.43 | 0.2 | 0.83 | 0.52 |
| 223 | R | 35 | 0.15 | 0.2 | 0.83 | 0.52 |
| 374 | M | 3 | 0.01 | 1.0 | 0.00 | 0.50 |
| 196 | R | 4 | 0.27 | 0.2 | 0.61 | 0.48 |
| 242 | M | 30 | 0.04 | 1.0 | 0.00 | 0.46 |
| 385 | M | 29 | 0.01 | 1.0 | 0.00 | 0.46 |
| 222 | K | 10 | 0.17 | 0.3 | 0.42 | 0.41 |
| 63 | M | 69 | 0.00 | 1.0 | 0.00 | 0.40 |
| 221 | M | 97 | 0.00 | 1.0 | 0.00 | 0.35 |
| 220 | M | 98 | 0.00 | 1.0 | 0.00 | 0.35 |
| 43 | H | 12 | 0.62 | 0.5 | 0.00 | 0.35 |
| 238 | W | 27 | 0.22 | 0.6 | 0.00 | 0.34 |
| 20 | H | 36 | 0.81 | 0.5 | 0.00 | 0.33 |
| 269 | H | 3 | 0.24 | 0.5 | 0.00 | 0.33 |
| 139 | H | 16 | 0.39 | 0.5 | 0.00 | 0.32 |
| 231 | H | 16 | 0.36 | 0.5 | 0.00 | 0.32 |
| 262 | H | 15 | 0.29 | 0.5 | 0.00 | 0.31 |
| 287 | H | 28 | 0.02 | 0.5 | 0.09 | 0.30 |
| 209 | H | 28 | 0.30 | 0.5 | 0.00 | 0.30 |
| 73 | H | 16 | 0.09 | 0.5 | 0.00 | 0.29 |
| 233 | K | 6 | 0.38 | 0.3 | 0.00 | 0.27 |
| 243 | K | 9 | 0.39 | 0.3 | 0.01 | 0.27 |
| 234 | K | 17 | 0.51 | 0.3 | 0.00 | 0.26 |
| 343 | K | 25 | 0.51 | 0.3 | 0.00 | 0.25 |
| 380 | K | 16 | 0.35 | 0.3 | 0.00 | 0.25 |
| 101 | R | 10 | 0.43 | 0.2 | 0.00 | 0.25 |
| 310 | K | 16 | 0.33 | 0.3 | 0.00 | 0.25 |
| 201 | K | 21 | 0.39 | 0.3 | 0.00 | 0.24 |
| 136 | K | 13 | 0.25 | 0.3 | 0.00 | 0.24 |
| 178 | R | 6 | 0.29 | 0.2 | 0.00 | 0.24 |
| 368 | K | 7 | 0.13 | 0.3 | 0.00 | 0.24 |
| 394 | K | 65 | 0.98 | 0.3 | 0.00 | 0.24 |
| 160 | Y | 73 | 0.19 | 0.5 | 0.00 | 0.23 |
| 217 | K | 44 | 0.55 | 0.3 | 0.00 | 0.23 |
| 69 | K | 42 | 0.47 | 0.3 | 0.00 | 0.22 |
| 365 | K | 28 | 0.19 | 0.3 | 0.00 | 0.21 |
| 174 | K | 40 | 0.37 | 0.3 | 0.00 | 0.21 |
| 129 | K | 53 | 0.56 | 0.3 | 0.00 | 0.21 |
| 194 | W | 99 | 0.01 | 0.6 | 0.00 | 0.21 |
| 163 | K | 41 | 0.34 | 0.3 | 0.00 | 0.21 |
| 187 | Y | 82 | 0.06 | 0.5 | 0.00 | 0.21 |
| 154 | K | 33 | 0.18 | 0.3 | 0.00 | 0.21 |
| 274 | K | 46 | 0.36 | 0.3 | 0.00 | 0.20 |
| 125 | K | 60 | 0.54 | 0.3 | 0.00 | 0.20 |
| 387 | R | 13 | 0.00 | 0.2 | 0.00 | 0.20 |
| 300 | K | 50 | 0.38 | 0.3 | 0.00 | 0.20 |
| 297 | Y | 94 | 0.15 | 0.5 | 0.00 | 0.20 |
| 38 | Y | 89 | 0.01 | 0.5 | 0.00 | 0.19 |
| 39 | R | 34 | 0.22 | 0.2 | 0.00 | 0.19 |
| 193 | K | 40 | 0.12 | 0.3 | 0.00 | 0.19 |
| 328 | K | 46 | 0.20 | 0.3 | 0.00 | 0.19 |
| 244 | Y | 98 | 0.00 | 0.5 | 0.00 | 0.18 |
| 138 | Y | 99 | 0.01 | 0.5 | 0.00 | 0.18 |
| 93 | H | 92 | 0.05 | 0.5 | 0.00 | 0.17 |
| 331 | K | 56 | 0.20 | 0.3 | 0.00 | 0.17 |
| 155 | K | 54 | 0.16 | 0.3 | 0.00 | 0.17 |
| 259 | K | 71 | 0.37 | 0.3 | 0.00 | 0.17 |
| 135 | K | 63 | 0.24 | 0.3 | 0.00 | 0.17 |
| 334 | H | 99 | 0.00 | 0.5 | 0.00 | 0.16 |
| 290 | K | 83 | 0.36 | 0.3 | 0.00 | 0.15 |
| 25 | K | 64 | 0.05 | 0.3 | 0.00 | 0.15 |
| 191 | K | 83 | 0.31 | 0.3 | 0.00 | 0.14 |
| 168 | K | 89 | 0.23 | 0.3 | 0.00 | 0.13 |
| 335 | K | 89 | 0.19 | 0.3 | 0.00 | 0.12 |

## Supplementary Table S4. Evolutionary residue-frequency distribution

Supplementary Table S4 Evolutionary residue-frequency distribution for Met55 in α-amylase AmyC derived from the HSSP alignment.

| **AA** | **Frequency_percent** | **Count_raw** | **Mutation_Risk** |
| --- | --- | --- | --- |
| A | 0 | 0 | 0.84 |
| C | 0 | 0 | 0.97 |
| D | 0 | 0 | 0.79 |
| E | 0 | 0 | 0.71 |
| F | 4 | 33 | 0.60 |
| G | 0 | 0 | 0.92 |
| H | 0 | 0 | 0.67 |
| I | 19 | 156 | 0.46 |
| K | 0 | 0 | 0.66 |
| L | 18 | 148 | 0.46 |
| M | 52 | 427 |  |
| N | 0 | 0 | 0.78 |
| P | 0 | 0 | 0.78 |
| Q | 0 | 0 | 0.70 |
| R | 0 | 0 | 0.68 |
| S | 0 | 0 | 0.84 |
| T | 1 | 8 | 0.72 |
| V | 6 | 49 | 0.57 |
| W | 0 | 0 | 0.94 |
| Y | 0 | 0 | 0.83 |

## Supplementary Table S5. Comparison of computational approaches

Supplementary Table S5 Comparison of computational approaches for oxidation site prediction in proteins

| **Tool (Reference)** | **Year** | **Target Residues** | **Input Required** | **Evolutionary Signal** | **Key Structural Features** | **Scope** | **Reported Performance** | **Substitution Guidance** | **Availability** |
| --- | --- | --- | --- | --- | --- | --- | --- | --- | --- |
| COPA [26] | 2008 | Cys only | Solved 3D structure | None | SASA, pKa, inter-Cys distance | General proteins | Acc = 80.1% (LOO-CV) | No | Web |
| Aledo et al. [27] | 2017 | Met only | Structure + proteomics | None | SASA, sequence context | General proteins | AUC = 0.85 | No | No |
| MetODeep [28] | 2019 | Met only | Sequence only | DNN implicit embeddings | None (sequence- based) | General proteins | AUC = 0.81 | No | Web |
| Mapes et al. [29] | 2019 | Cys only | Sequence (+ optional 3D) | PSSM (local window) | SASA, pKa, RAMmod (3D dist.) | General proteins | AUC = 0.85 (BALOSCTdb) | No | GitHub |
| Veredas et al. [30] | 2020 | Met only | Sequence + 3D | None | SASA, structural context | General proteins | AUC = 0.83 | No | No |
| Tavella et al. [31] | 2022 | Met only | 3D structure + MD simulation | None | WCN-OH (water + –OH groups within 6 Å) | mAbs only | Sensitivity = 0.88 | No | No |
| **EvoMut** (this work) | 2026 | M, C, W, Y, H, K, R | PDB + HSSP (MSA) | Full MSA (HSSP, whole- protein) | RSA, B-factor, metal proximity | General proteins | Case-validated (4 proteins) | Yes (+ feasibility score) | Web |

**Notes:** LOO-CV = leave-one-out cross-validation. SASA = solvent-accessible surface area. pKa = calculated ionization constant. PSSM = position-specific scoring matrix (local sequence window). MSA = multiple sequence alignment. HSSP = Homology-derived Secondary Structure of Proteins database. WCN-OH = water coordination number with neighboring hydroxyl groups. mAbs = monoclonal antibodies. MD = molecular dynamics simulation. EvoMut performance is based on retrospective case-study validation (A1AT, AmyA, AmyC, VP); prospective experimental validation is a subject of future work.

## Supplementary Table S6. Chemical susceptibility weights for hydrogen peroxide and peroxyl radicals (ROO•), listed separately

Supplementary Table S6 Chemical susceptibility weights and supporting rate data for hydrogen peroxide and peroxyl radicals (ROO•), listed separately.

|  | **Met** | **Cys (free)** | **Trp** | **Tyr** | **His** | **Lys** | **Arg** |
| --- | --- | --- | --- | --- | --- | --- | --- |
| H₂O₂ weight | 0.47 | 1.00 | 0.00 | 0.00 | 0.00 | 0.00 | 0.00 |
| H₂O₂ direct k (M⁻¹s⁻¹) | ~0.1 | ~17 | ~0 | ~0 | ~0 | ~0 | ~0 |
| ROO• weight | 0.90 | 1.00 | 0.65 | 0.55 | 0.30 | 0.10 | 0.10 |
| ROO• consumption rank | 2nd | 1st | 3rd | 4th | 5th | – | – |

## Supplementary Table S7. Chemical susceptibility weights for hypochlorous acid (HOCl)

Supplementary Table S7 Chemical susceptibility weights and HOCl second-order rate constants for hypochlorous acid (HOCl).

|  | **Met** | **Cys (free)** | **His** | **Trp** | **Lys** | **Tyr** | **Arg** | **Cys-SS** |
| --- | --- | --- | --- | --- | --- | --- | --- | --- |
| k (M⁻¹s⁻¹) | 3.8×10⁷ | 3.0×10⁷ | 1.0×10⁵ | 1.1×10⁴ | 5.0×10³ | 44 | 26 | 1.6×10⁵ |
| EvoMut weight | 0.86 | 1.00 | 0.50 | 0.55 | 0.32 | 0.35 | 0.00 | 0.05 |

## Supplementary Table S8. Chemical susceptibility weights for metal-catalyzed oxidation (MCO / •OH)

Supplementary Table S8 Chemical susceptibility weights and target roles for metal-catalyzed oxidation (MCO / •OH).

|  | **His** | **Lys** | **Arg** | **Cys** | **Met** | **Trp** | **Tyr** |
| --- | --- | --- | --- | --- | --- | --- | --- |
| EvoMut weight | 1.00 | 0.85 | 0.80 | 0.60 | 0.35 | 0.20 | 0.20 |
| MCO role | Primary (→2-oxo-His) | Primary (→AAS) | Primary (→GS) | Secondary | Secondary | Minor | Minor |

## Supplementary Table S9. Chemical susceptibility weights for singlet oxygen (¹O₂) and UV photo-oxidation, listed separately

Supplementary Table S9 Chemical susceptibility weights for singlet oxygen (¹O₂) and UV photo-oxidation, listed separately.

|  | **Trp** | **His** | **Tyr** | **Met** | **Cys** | **Lys** | **Arg** |
| --- | --- | --- | --- | --- | --- | --- | --- |
| ¹O₂ weight | 0.95 | 1.00 | 0.65 | 0.85 | 0.60 | 0.00 | 0.00 |
| ¹O₂ total k (×10⁷ M⁻¹s⁻¹) | 9 | 17 | ~2 | 3 | ~0.5 | ~0 | ~0 |
| ¹O₂ reactive fraction(%) | ~65 | ~35 | ~40 | ~30 | ~50 | – | – |
| UV weight (free) | 1.00 | 0.25 | 0.45 | 0.05 | 0.10 | 0.00 | 0.00 |
| UV basis | 280 nm chromophore | abs. | abs. | indirect | S–S 0.40 | – | – |

## Supplementary Table S10. Oxidant penetration tiers

Supplementary Table S10 Oxidant penetration tiers used to assign the solvent-exposure weight (w_E_).

| **Tier** | **Oxidant** | **w_E_** | **Rationale** | **Key evidence** |
| --- | --- | --- | --- | --- |
| High surface- dependence | **HOCl** | **0.15** | Large (MW=52), negatively charged at physiological pH (pKa=7.53 → OCl⁻ at pH 7.4), high polarity; cannot partition into hydrophobic core | *Pattison & Davies (2001): HOCl reactivity strongly correlated with surface exposure in intact proteins; minimal modification of buried residues in globular proteins* |
| Standard | Peroxyl radicals (ROO•) | **0.10** | Medium-sized diffusible radical; standard surface dependence | *Default calibrated against A1AT and α-amylase case studies* |
| Low surface- dependence | H₂O₂, MCO (•OH), ¹O₂, UV | **0.05** | H₂O₂: MW=34, neutral, traverses aquaporin channels; ¹O₂: τ~3 μs, diffusion radius ~200 nm; UV photons: no mass, mean free path >> protein diameter; •OH: generated in situ | *Sjöberg et al. (2018): H₂O₂ oxidizes buried Cys in peroxiredoxins; Gracanin et al. (2009): ¹O₂ oxidizes buried Trp/His; (1990): •OH reacts within 1–2 nm of metal center* |

## Supplementary Table S11. Per-species ORI term weights and redistribution

Supplementary Table S11 Per-species ORI term weights (w_E_, w_C_, w_S_, w_K_) and their derivation basis, including the computed screening (aggregate) default used when the oxidant is unspecified.

| **Species** | **w_E_** | **w_C_** | **w_S_** | **w_K_** | **basis** |
| --- | --- | --- | --- | --- | --- |
| H₂O₂ | 0.05 | 0.45 | 0.35 | 0.15 | log-k |
| ROO• (peroxyl) | 0.10 | 0.35 | 0.40 | 0.15 | consumption-rank |
| HOCl | 0.15 | 0.40 | 0.30 | 0.15 | log-k |
| MCO / •OH | 0.05 | 0.30 | 0.50 | 0.15 | carbonyl-yield |
| ¹O₂ | 0.05 | 0.55 | 0.25 | 0.15 | log-k |
| UV | 0.05 | 0.55 | 0.25 | 0.15 | chromophore-absorption |
| Screening (aggregate default) | 0.07 | 0.44 | 0.34 | 0.15 | computed |

# **Supplementary Data Availability**

Complete EvoMut analysis outputs, including structural input files, HSSP alignments, configuration files, and detailed analysis results, are available in the Zenodo repository:

<https://doi.org/10.5281/zenodo.19039897>

Each case study described in the manuscript is organized in a separate directory containing the input data and EvoMut output files used in the analysis.

# **References:**

1.	Amici A, Levine RL, Tsai L, Stadtman ER. Conversion of amino acid residues in proteins and amino acid homopolymers to carbonyl derivatives by metal-catalyzed oxidation reactions. J Biol Chem. 1989;264: 3341–3346.

2.	Regino CAS, Richardson DE. Bicarbonate-catalyzed hydrogen peroxide oxidation of cysteine and related thiols. Inorganica Chim Acta. 2007;360: 3971–3977.

3.	Winterbourn CC, Metodiewa D. Reactivity of biologically important thiol compounds with superoxide and hydrogen peroxide. Free Radic Biol Med. 1999;27: 322–328.

4.	Sjöberg B, Foley S, Cardey B, Fromm M, Enescu M. Methionine oxidation by hydrogen peroxide in peptides and proteins: A theoretical and Raman spectroscopy study. J Photochem Photobiol B. 2018;188: 95–99.

5.	Davies MJ. Protein oxidation and peroxidation. Biochem J. 2016;473: 805–825.

6.	Dorta E, Ávila F, Fuentes-Lemus E, Fuentealba D, López-Alarcón C. Oxidation of myofibrillar proteins induced by peroxyl radicals: Role of oxidizable amino acids. Food Res Int. 2019;126: 108580.

7.	Folzer E, Diepold K, Bomans K, Finkler C, Schmidt R, Bulau P, et al. Selective oxidation of methionine and tryptophan residues in a therapeutic IgG1 molecule. J Pharm Sci. 2015;104: 2824–2831.

8.	Leinisch F, Mariotti M, Rykaer M, Lopez-Alarcon C, Hägglund P, Davies MJ. Peroxyl radical- and photo-oxidation of glucose 6-phosphate dehydrogenase generates cross-links and functional changes via oxidation of tyrosine and tryptophan residues. Free Radic Biol Med. 2017;112: 240–252.

9.	Fuentes-Lemus E, Silva E, Barrias P, Aspee A, Escobar E, Lorentzen LG, et al. Aggregation of α- and β- caseins induced by peroxyl radicals involves secondary reactions of carbonyl compounds as well as di-tyrosine and di-tryptophan formation. Free Radic Biol Med. 2018;124: 176–188.

10.	Pattison DI, Davies MJ. Absolute rate constants for the reaction of hypochlorous acid with protein side chains and peptide bonds. Chem Res Toxicol. 2001;14: 1453–1464.

11.	Storkey C, Davies MJ, Pattison DI. Reevaluation of the rate constants for the reaction of hypochlorous acid (HOCl) with cysteine, methionine, and peptide derivatives using a new competition kinetic approach. Free Radic Biol Med. 2014;73: 60–66.

12.	Pattison DI, Hawkins CL, Davies MJ. Hypochlorous acid-mediated protein oxidation: how important are chloramine transfer reactions and protein tertiary structure? Biochemistry. 2007;46: 9853–9864.

13.	Choe JK, Richards DH, Wilson CJ, Mitch WA. Degradation of amino acids and structure in model proteins and bacteriophage MS2 by chlorine, bromine, and ozone. Environ Sci Technol. 2015;49: 13331–13339.

14.	Peskin AV, Winterbourn CC. Kinetics of the reactions of hypochlorous acid and amino acid chloramines with thiols, methionine, and ascorbate. Free Radic Biol Med. 2001;30: 572–579.

15.	Stadtman ER. Metal ion-catalyzed oxidation of proteins: biochemical mechanism and biological consequences. Free Radic Biol Med. 1990;9: 315–325.

16.	Schöneich C. Mechanisms of metal-catalyzed oxidation of histidine to 2-oxo-histidine in peptides and proteins. J Pharm Biomed Anal. 2000;21: 1093–1097.

17.	Requena JR, Chao CC, Levine RL, Stadtman ER. Glutamic and aminoadipic semialdehydes are the main carbonyl products of metal-catalyzed oxidation of proteins. Proc Natl Acad Sci U S A. 2001;98: 69–74.

18.	Matheson IB, Etheridge RD, Kratowich NR, Lee J. The quenching of singlet oxygen by amino acids and proteins. Photochem Photobiol. 1975;21: 165–171.

19.	Michaeli A, Feitelson J. Reactivity of singlet oxygen toward amino acids and peptides. Photochem Photobiol. 1994;59: 284–289.

20.	Dyer JM, Bringans SD, Bryson WG. Characterisation of photo-oxidation products within photoyellowed wool proteins: tryptophan and tyrosine derived chromophores. Photochem Photobiol Sci. 2006;5: 698–706.

21.	Pattison DI, Rahmanto AS, Davies MJ. Photo-oxidation of proteins. Photochem Photobiol Sci. 2012;11: 38–53.

22.	Gracanin M, Hawkins CL, Pattison DI, Davies MJ. Singlet-oxygen-mediated amino acid and protein oxidation: formation of tryptophan peroxides and decomposition products. Free Radic Biol Med. 2009;47: 92–102.

23.	Boreen AL, Edhlund BL, Cotner JB, McNeill K. Indirect photodegradation of dissolved free amino acids: the contribution of singlet oxygen and the differential reactivity of DOM from various sources. Environ Sci Technol. 2008;42: 5492–5498.

24.	Zhao Z, Poojary MM, Skibsted LH, Lund MN. Cleavage of disulfide bonds in cystine by UV-B illumination mediated by tryptophan or tyrosine as photosensitizers. J Agric Food Chem. 2020;68: 6900–6909.

25.	Abaskharon RM, Gai F. Direct measurement of the tryptophan-mediated photocleavage kinetics of a protein disulfide bond. Phys Chem Chem Phys. 2016;18: 9602–9607.

26.	Sanchez R, Riddle M, Woo J, Momand J. Prediction of reversibly oxidized protein cysteine thiols using protein structure properties. Protein Sci. 2008;17: 473–481.

27.	Aledo JC, Cantón FR, Veredas FJ. A machine learning approach for predicting methionine oxidation sites. BMC Bioinformatics. 2017;18: 430.

28.	Lopez-Garcia G, Jerez JM, Urda D, Veredas FJ. MetODeep: A deep learning approach for prediction of methionine oxidation sites in proteins. 2019 International Joint Conference on Neural Networks (IJCNN). IEEE; 2019. doi:10.1109/ijcnn.2019.8851901

29.	Mapes NJ Jr, Rodriguez C, Chowriappa P, Dua S. Residue adjacency matrix based feature engineering for predicting cysteine reactivity in proteins. Comput Struct Biotechnol J. 2019;17: 90–100.

30.	Veredas FJ, Urda D, Subirats JL, Cantón FR, Aledo JC. Combining feature engineering and feature selection to improve the prediction of methionine oxidation sites in proteins. Neural Comput Appl. 2020;32: 323–334.

31.	Tavella D, Ouellette DR, Garofalo R, Zhu K, Xu J, Oloo EO, et al. A novel method for in silico assessment of Methionine oxidation risk in monoclonal antibodies: Improvement over the 2-shell model. PLoS One. 2022;17: e0279689.
